# Spatiotemporal Evolution and Influencing Factors of Carbon Sink Dynamics at County Scale: A Case Study of Shaanxi Province, China

**DOI:** 10.3390/ijerph182413081

**Published:** 2021-12-11

**Authors:** Shuohua Liu, Xiao Zhang, Yifan Zhou, Shunbo Yao

**Affiliations:** 1 College of Economics and Management, Northwest A&F University, Xianyang 712100, China; ShuohuaLiu@nwafu.edu.cn (S.L.); zhangxiao96@nwafu.edu.cn (X.Z.); 2 Center for Resource Economics and Environment Management, Northwest A&F University, Xianyang 712100, China; 3 College of Economics and Management, Hebei Agricultural University, Baoding 071000, China; fanzhou0930@126.com

**Keywords:** carbon sinks, spatiotemporal evolution, ESDA, spatial panel Durbin model, driving factors

## Abstract

To explore the spatiotemporal evolution of carbon sinks in Shaanxi Province, and their impact mechanisms, this study used panel data from 107 counties (districts) in Shaanxi Province from 2000 to 2017. First, we conducted spatial distribution directional analysis and exploratory spatial data analysis (ESDA). Then, we constructed a geographic spatial weight matrix and used the spatial panel Durbin model to analyze the driving factors of carbon sink changes in Shaanxi Province, from the perspective of spatial effects. The results showed that: (1) The temporal evolution of carbon sinks during the study period showed an overall upward trend, but the carbon sinks of counties (districts) differed greatly, and the center of gravity of carbon sinks, as a whole, showed the characteristics of “south to north” migration. (2) The carbon sinks of Shaanxi Province have a significant positive global spatial autocorrelation in geographic space. The local spatial pattern was characterized by low-value agglomeration (low-low cluster) and high-value agglomeration (high-high cluster), supplemented by high-value bulge (high-low outlier) and low-value collapse (low-high outlier). (3) The result of the spatial measurement model proved that the spatial Durbin model, with dual fixed effects of time and space, should be selected. In the model results, factors such as population, per capita gross domestic product (GDP), local government general budget expenditure, and local government general budget revenue all reflect strong spatial spillover effects. Accordingly, in the process of promoting “carbon neutrality”, the government needs to comprehensively consider the existence of spatial spillover effects between neighboring counties (districts), and strengthen the linkage-management and control roles of counties (districts) in increasing carbon sinks.

## 1. Introduction

The Paris Agreement, signed at the Paris Climate Conference, set a goal of limiting the global average temperature rise to within 2 °C by controlling carbon emissions [1]. The recent summit, Cop 26, in Glasgow, also proposed a goal; to “secure global net zero by mid-century and keep 1.5 degrees within reach” [2]. Countries are being asked to come forward with ambitious 2030 emission reduction targets that align with reaching net zero by the middle of the century. Additionally, countries need to accelerate the phase-out of coal, curtail deforestation, protect and restore ecosystems, and build collaboration between governments, businesses, and civil society to achieve these goals. Facing the problems brought by the urgent mitigation of climate change and the upgrading of industrial structure, China has made a commitment to the world to achieve carbon neutrality by 2060, before reaching the peak of carbon emissions [3]. In a 2021 government report, “carbon peaking and carbon neutrality” was listed as one of the key tasks this year [4]. This means that China’s carbon emissions will be zeroed in 2060 [5], and the traditional energy structure will face major changes. Among them, the proportion of the fossil energy industry will decrease, and the proportion of clean energy, such as photovoltaics and wind energy, will increase significantly. Moreover, the country should increase the popularity of electric vehicles and transition to the goal of zero-emission vehicles [2], as well as encourage investment in renewable energy, promote the proportion of green energy in China, and promote high-quality economic development.

From the perspective of many European developed countries, the time interval between China’s carbon peak and its carbon neutrality target is relatively short, which requires us to consider increasing carbon sinks while limiting carbon emissions. However, controlling carbon emissions and increasing carbon sinks will limit the speed of China’s economic development [6]. Therefore, it is very necessary to explore methods of achieving the goal of “dual carbon” at the lowest cost. The nature-based approach is currently the most cost-effective way to increase carbon sinks, and at the same time promotes the high-quality development of forests [7]. However, as a national policy goal, “carbon neutrality” relies heavily on policy support and government investment. In addition, it may limit the economic development of local and neighboring regions. Therefore, analyzing the influencing factors of carbon sinks at the county level is related to government decision-making and the coordinated development of various regions.

Research on the impact mechanism of carbon sinks mostly focuses on the perspective of natural science under the forestry field [8]. Based on small-scale observations, these studies investigate the impact of forest stand structure, stand density [9], atmospheric nitrogen deposition [10], and forest management [11,12] on carbon sink levels [13]. This type of research mostly uses simply measures, and describes the impact of these factors on carbon sinks in a small range; there is a possibility of reduced accuracy in a large range. Large-scale research fields are more extensive. From a macro perspective, researchers use the InVEST model [14,15] to investigate the relationship, and results of interaction, between carbon sinks and climate change [16], land use change [17,18], landscape design [19], and carbon footprint [20]. However, due to their spatial relevance and heterogeneity, existing studies have not incorporated spatial factors into carbon sink impact mechanism research.

In view of this, this paper drew on existing research and collected panel data on the socio-economic status and carbon sinks of 107 counties (districts) in Shaanxi Province from 2000 to 2017. We used the exploratory spatial data analysis (ESDA) method to analyze the global and local pattern characteristics of carbon sinks, and explore the spatiotemporal evolution trends of carbon sinks in various counties (districts) in Shaanxi Province. Then, the spatial Durbin model was used to parametrically estimate the spatial spillover effects of carbon sinks, and the partial differential decomposition method was used to decompose and analyze the spatial effects of various driving factors of carbon sinks in Shaanxi Province. This was expected to provide suggestions for increasing carbon sinks between counties (districts) and promote the realization of the regional “carbon neutral” goal.

## 2. Study Area, Data and Methods

### 2.1. Study Area

Shaanxi Province is located in the hinterland of western China, between 105°29′ E–111°15′ E, 31°42′ N–39°35′ N. As the Qinling Mountains on the boundary between the north and south of China traverse the entire province, the climate between the north and the south of Shaanxi Province is quite different [21]. Along the Great Wall in northern Shaanxi, which is a semi-arid region, there is a moderately temperate climate. Guanzhong and most parts of northern Shaanxi have a warm temperate climate, and southern Shaanxi has a northern subtropical climate. The terrain is generally high in the north and south, and low in the middle. The average annual precipitation is 340–1240 mm, and the average annual temperature is 7–16 °C. Both precipitation and temperature decrease from south to north [22]. Natural geographical factors affect the area of forest coverage and the distribution area of forest species. The southern part of Shaanxi Province is dominated by economic forests and natural forests, and there are many artificial ecological forests in the north, to provide the functions of windbreak and sand fixation, as well as soil and water conservation. As one of the main bodies of carbon sequestration, forests are closely related to the distribution of carbon sinks [23,24].

**Figure 1:**
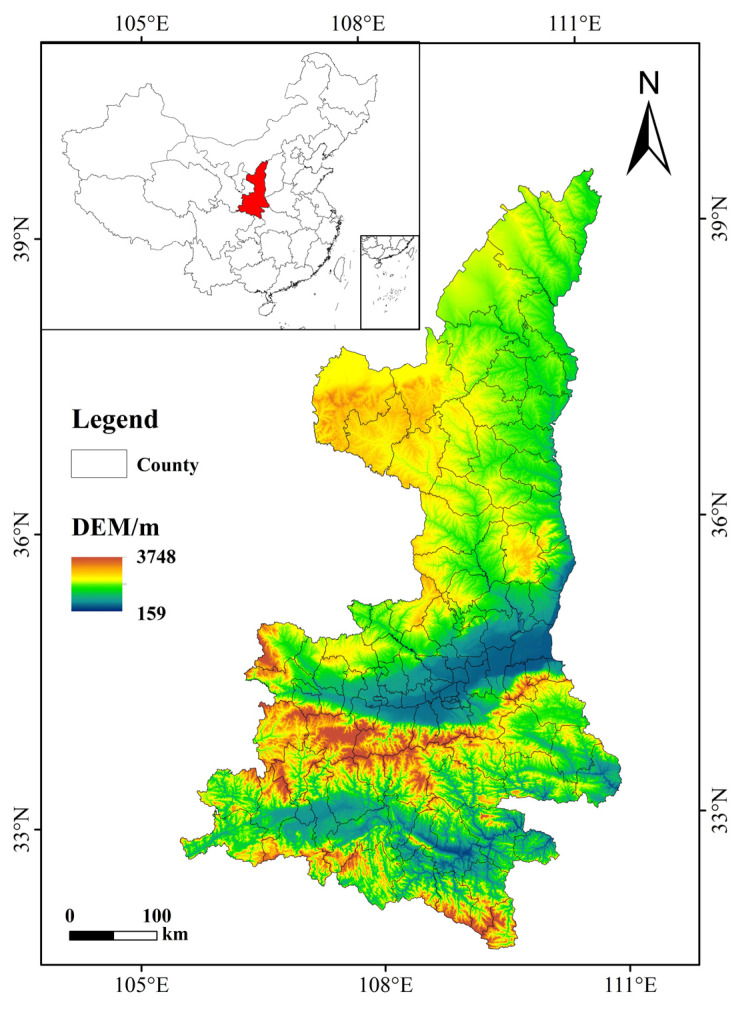
Study area.

To cope with climate change and promote implementation of “carbon peaking and carbon neutrality”, Shaanxi Province is actively responding to the two aspects of carbon emissions and carbon sinks. On the one hand, by adjusting industrial structure, optimizing energy structure, promoting green transportation, and building green buildings. On the other hand, by developing green agriculture, continuing to increase green agriculture, and promoting bioenergy with carbon capture and storage (BECCS) pilot demonstrations [25]. A response system has been formed that includes pilot norms in multiple fields, extensive social participation, and gradual improvements in systems and mechanisms. The location map of Shaanxi Province is shown in Figure 1.

### 2.2. Variable Selection and Data Sources

#### 2.2.1. Variable Selection

To study the spatiotemporal evolution and influencing factors of carbon sinks at the county scale, this paper takes the carbon sinks of each county (district) as the explained variable. When selecting influencing factors, considering that carbon peaking and carbon neutrality are closely related to social and economic development, our model mainly examines the impact of social and economic factors in counties (districts) on carbon sinks.

First, considering that the increase in carbon sinks in counties (districts) mainly comes from woodlands and bodies of water, the increase in carbon sinks in grassland and arable land is relatively small. In areas with more woodland and water, the scope of human life is smaller, so there should be a negative correlation between population and carbon sinks. On the other hand, although the increase in carbon sinks requires a large amount of financial investment, it will also drive local employment. For example, the advancement of carbon sink afforestation, the Sloping Land Conversion Program (SLCP), and the Natural Forest Protection Project (NFPP) have created a large number of employment opportunities. Moreover, forest maintenance measures, such as thinning, have brought a large amount of timber output and income, and promote the development of downstream companies related to wood products; the maintenance of bodies of water and forests will bring part of the tourism income, and stimulate the economic development of the region. However, it may lead to the outflow of labor into neighboring areas, and the slowdown of economic development. Therefore, in the spatial measurement model, the impact of per capita GDP (agdp) on carbon sinks should be examined.

Secondly, measures to increase carbon sinks, such as afforestation, tending, and forest quality improvement, are highly dependent on local fiscal revenues and expenditures. Therefore, this paper takes local government general budget revenue (income) and local government general budget expenditure (outcome) into the consideration range of the spatial measurement model. Additionally, to intuitively reflect the consumption demand in the region, measure the level of regional development, and reflect the impact of economic prosperity on carbon sinks, this paper incorporates the total retail sales of consumer goods in each county (district) into the model. To reduce possible heteroscedasticity in the model, the three variables of county (district) income, outcome, and retail are taken using logarithms, which are recorded as lnincome, lnoutcome, and lnretail.

#### 2.2.2. Data Sources

The carbon sink data comes from the “scientific data” database (https://doi.org/10.1038/s41597-020-00736-3, accessed on 2 July 2021). This data was the vegetation net primary productivity (NPP), calculated based on the MOD17A3 product, provided by the National Aeronautics and Space Administration (NASA), multiplied by the conversion factor. The land-vegetation carbon sink value was then calculated based on the previously studied carbon sink conversion coefficient (1.62/0.45). Data included evergreen needleleaf forest (ENF), evergreen broadleaf forest (EBF), deciduous needleleaf forest (DNF), deciduous broadleaf forest (DBF), mixed forests (MF), closed shrublands (CShrub), open shrublands (OShrub), woody savannas (WSavanna), savannas (Savanna), grasslands (Grass), and croplands (Crop) [26]. Finally, the county (district) carbon sink value was obtained by cutting through the 2015 county-level vector diagram.

Socioeconomic data: first, the per capita GDP and population data of the counties (districts) in Shaanxi Province were all from the “Shaanxi Statistical Yearbook”, “Shaanxi County Statistical Yearbook” and “Shaanxi Regional Statistical Yearbook”. Second, the data of 107 administrative boundaries in Shaanxi Province came from the basic geographic database (http://www.webmap.cn, accessed on 5 July 2021), and the area of each county (district) was calculated through the administrative boundary data.

**Figure 2:**
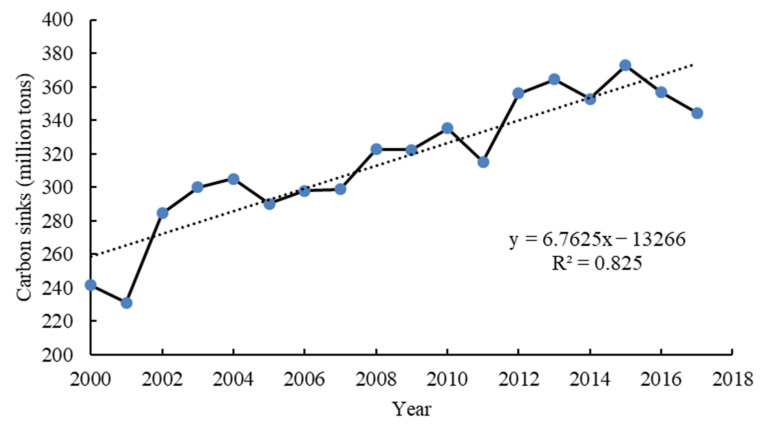
Temporal evolution of carbon sinks in Shaanxi Province.

**Figure 3:**
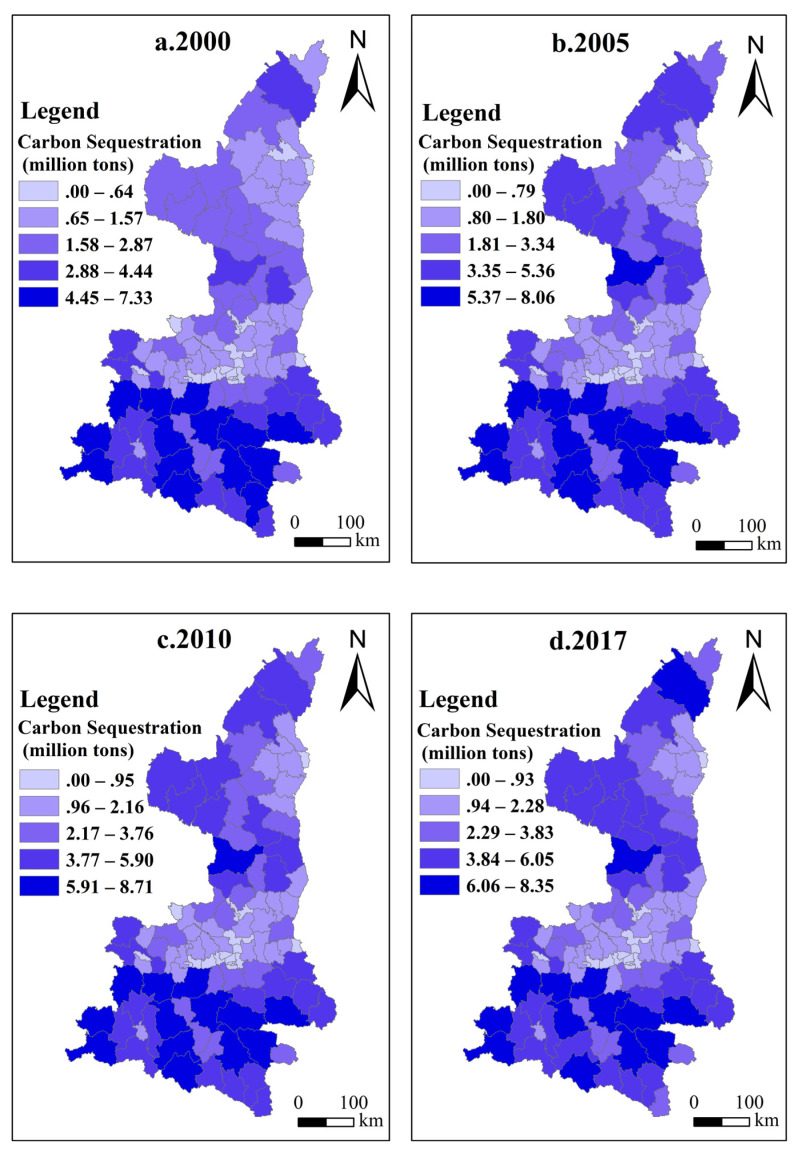
The spatiotemporal evolution of carbon sequestration in Shaanxi Province. (**a**) Carbon sequestration in 2000; (**b**) carbon sequestration in 2005; (**c**) carbon sequestration in 2010; (**d**) carbon sequestration in 2017.

**Figure 4:**
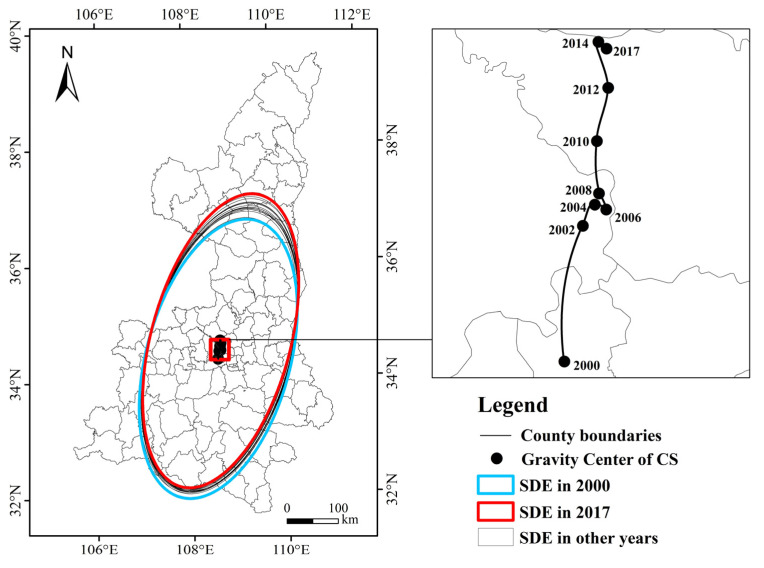
SDE and center-of-gravity migration path.

**Figure 5:**
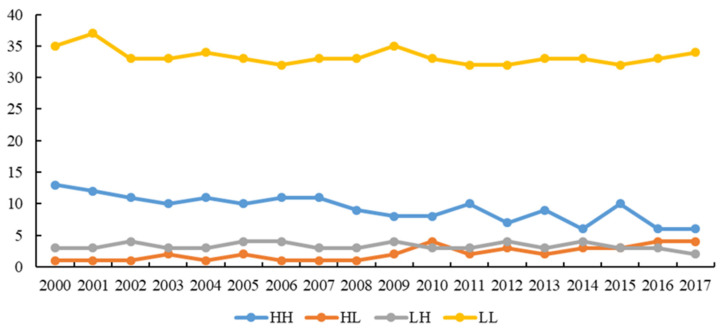
The evolution characteristics of the local spatial pattern of carbon sinks.

Figure 1 uses ArcGIS 10.7 software and data from the Resource and Environmental Science and Data Center of the Chinese Academy of Sciences (http://www.resdc.cn/, accessed on 6 July 2021). Figures 2 and 5 use Excel and data from the “scientific data”. Figures 3 and 4 use ArcGIS 10.7 software (Esri, Redlands, CA, USA) and data from the “scientific data”. Tables 1–6 use MATLAB software (MathWorks Corporation, Middlesex, MA, USA) and data from the “Shaanxi Statistical Yearbook”, “Shaanxi County Statistical Yearbook”, “Shaanxi Regional Statistical Yearbook”, and “scientific data”.

### 2.3. Research Methods

#### 2.3.1. Analysis of Spatial Distribution Directionality

The spatial distribution directional analysis refers to the outline and dominant direction of the observed variable in the spatial distribution. Standard deviation ellipse (SDE) is a spatial statistical method used to reveal the spatial distribution characteristics of elements. This method mainly uses the measurement and calculation of the center of gravity, major axis, minor axis, azimuth angle, and other parameters of the spatial distribution ellipse of geographic elements to quantitatively describe the spatial distribution characteristics of the observed variables in the study area. The definition formula is as follows:

(1) $$\mathrm{Center}\;\mathrm{of}\;\mathrm{gravity}\;\mathrm{coordinates}\text{:}\;{\bar{X}}_{w}=\sum_{i=1}^{n}w_{i}x_{i}/\sum_{i=1}^{n}w_{i};{\bar{Y}}_{w}=\sum_{i=1}^{n}w_{i}y_{i}/\sum_{i=1}^{n}w_{i}$$

(2) $$\tan\theta =\frac{\left(\sum_{i=1}^{n}w_{i}^{2}{\tilde{x}}_{i}^{2}-\sum_{i=1}^{n}w_{i}^{2}{\tilde{y}}_{i}^{2}\right)+\sqrt{{\left(\sum_{i=1}^{n}w_{i}^{2}{\tilde{x}}_{i}^{2}-\sum_{i=1}^{n}w_{i}^{2}{\tilde{y}}_{i}^{2}\right)}^{2}+4\sum_{i=1}^{n}w_{i}^{2}{\tilde{x}}_{i}^{2}{\tilde{y}}_{i}^{2}}}{2\sum_{i=1}^{n}w_{i}^{2}{\tilde{x}}_{i}{\tilde{y}}_{i}}$$

(3) $$x\text{-}\mathrm{axis}\;\mathrm{standard}\;\mathrm{deviation}\text{:}\;\sigma_{x}=\sqrt{\sum_{i=1}^{n}{\left(w_{i}{\tilde{x}}_{i}\cos\theta -w_{i}{\tilde{y}}_{i}\sin\theta \right)}^{2}/\sum_{i=1}^{n}w_{i}^{2}}$$

(4) $$y\text{-}\mathrm{axis}\;\mathrm{standard}\;\mathrm{deviation}\text{:}\;\sigma_{y}=\sqrt{\sum_{i=1}^{n}{\left(w_{i}{\tilde{x}}_{i}\sin\theta -w_{i}{\tilde{y}}_{i}\cos\theta \right)}^{2}/\sum_{i=1}^{n}w_{i}^{2}}$$

In the above formula: ${\bar{X}}_{w}$ and ${\bar{Y}}_{w}$ represent the weighted average center of each observation variable; (*x_i_*, *y_i_*) represents the spatial coordinates of the observed variable; *w_i_* represents the spatial weight; *θ* is the azimuth angle of the SDE, that is, the main trend direction of the data distribution; $\sigma_{x}$ and $\sigma_{y}$, respectively, represent the standard deviation of the x-axis and y-axis of the ellipse; ${\tilde{x}}_{i}$ and ${\tilde{y}}_{i}$, respectively, represent the coordinate deviation of each observed variable to the weighted average center.

#### 2.3.2. Global Moran’s I

Global spatial autocorrelation was used to explore the overall spatial correlation degree and significance of carbon sinks. Global Moran’s I is the most widely used spatial autocorrelation statistic, which was chosen to test the spatial attributes of carbon sinks in Shaanxi Province [27]. The spatial weight selects a weight matrix based on geographic distance, and the specific calculation formula is as follows:

(5) $$\mathrm{Moran}’s\;I=\frac{N}{S_{0}}\frac{\sum_{i=1}^{N}\sum_{j=1}^{N}w_{ij}\left(y_{i}-\bar{y}\right)\left(y_{i}-\bar{y}\right)}{\sum_{i}^{N}{\left(y_{i}-\bar{y}\right)}^{2}}$$

In the above formula, *N* represents the number of spatial units (the number of observations); *S*_0_ represents the sum of all elements of the spatial weight matrix; *y_i_* represents the observation value of the variable *y* in the spatial unit *i*; *w_ij_* represents the element in the spatial weight matrix; and the definition formula of *S*_0_ is:

(6) $$S_{0}=\overset{N}{\underset{i=1}{\sum }}\overset{N}{\underset{j=1}{\sum }}w_{ij}$$

The value range of Moran’s I statistic is [−1, 1]. A positive value represents a positive spatial autocorrelation, or spatial agglomeration phenomenon, and a negative value represents a negative spatial autocorrelation, or spatial dispersion [28]. A value of 1 indicates complete spatial agglomeration, a value of 0 indicates a random distribution in space, and a value of −1 indicates complete spatial dispersion.

#### 2.3.3. Local Moran’s I

Local autocorrelation statistics can decompose the global spatial autocorrelation Moran’s I statistics, and can be used to discover the contribution of each spatial unit observation value [29]. The definition formula of local Moran’s I statistic is:

(7) $$Local\;Moran’s\;I=\frac{y_{i}-\bar{y}}{S_{i}^{2}}\overset{N}{\underset{j=1,j\neq i}{\sum }}w_{ij}\left(y_{i}-\bar{y}\right)\;$$

$S_{i}^{2}$ is defined as:

(8) $$S_{i}^{2}=\frac{\sum_{j=1,j\neq i}^{N}w_{ij}}{N-1}-{\bar{y}}^{2}\;$$

#### 2.3.4. Spatial Durbin Model

##### Spatial Weight Matrix

Based on the above analysis of global and local autocorrelation, there are different degrees of regional correlation in carbon emissions among different counties in Shaanxi Province. Therefore, ordinary econometric models cannot investigate and deal with the analysis of spatial interaction (spatial autocorrelation) and spatial structure (spatial inhomogeneity). Therefore, according to the first law of geography, the relationship between regions will decrease with the increase of geographic distance, thus constructing a spatial weight matrix [30,31].

The paper used ArcGIS 10.7 software to construct a spatial weight matrix, and then used MATLAB software to convert it into a standardized spatial weight matrix in the county (district) format of 107 × 107, which was used as a model of spatial econometrics. The form is as follows:

(9) $$W=\left[\begin{array}{cccc} W_{11} & W_{12} & \cdots & W_{1n}\\ W_{21} & W_{22} & \ldots & W_{2n}\\ \vdots & \vdots & & \vdots \\ W_{n1} & W_{n2} & \ldots & W_{nn} \end{array}\right]\;$$

##### Spatial Durbin Model

Spatial measurement model refers to the processing of spatial interaction (spatial autocorrelation) and spatial unevenness of panel data in the regression model [32]. Commonly used spatial panel measurement models include SLM, SEM, and SDM. Spatial Durbin model is a general form of a spatial error and spatial lag model. The spatial panel Durbin model incorporates both explanatory variables and the spatial effects of explanatory variables into the econometric model setting, and also includes endogenous interaction effects (WY) and exogenous interaction effects (WX) [33,34]. Considering that carbon sinks and their influencing factors have strong spatial correlation, this paper chose the spatial Durbin model to estimate. The code used in the spatial Durbin model comes from Elhorst’s spatial econometrics MATLAB toolbox. The model data in this paper is the panel data of 107 counties (districts) in Shaanxi Province from 2000 to 2017.

(10) $$carbonsink_{it}=\rho Wy_{it}+X_{it}\beta +WX_{it}\theta +\varepsilon_{it}\;$$

In the above formula, *carbonsink_it_* is the observed value of the explained variable carbon sink; *X_it_* is the observed value of the explanatory variable, and in this paper is the various influencing factors that affect the change of the explained variable; *ρ* is the spatial regression coefficient of the explained variable; *θ* is the spatial regression coefficient of the explanatory variable; *β* is the regression coefficient of the explanatory variable; *ε_it_* is a random error term that obeys independent and identical distribution, indicating other factors that are not included in the measurement model; *W* is the spatial weight matrix constructed by the above method.

## 3. Results and Analysis

### 3.1. Spatiotemporal Analysis of Carbon Sequestration

To reflect the differences in carbon sinks of different counties (districts) in each year, in the spatial scope, the spatiotemporal evolution of carbon sinks in 107 counties (districts) in Shaanxi Province during the study period was comprehensively sorted out. First, we use Excel to draw a graph of the temporal evolution of carbon sinks (Figure 2). Then, we use ArcGIS 10.7 to connect with the county (district) location, select the natural break point method to classify the carbon sinks in 2000, 2005, 2010, and 2017, and visualize them (Figure 3a–d).

The temporal changes of carbon sinks in Shaanxi Province showed an overall upward trend, in Figure 2. The total carbon sink amounts in 2000, 2005, 2010, and 2017 were 241.4398, 290.0709, 335.2216, and 344.5110 million tons, respectively. In 2017, carbon sinks increased by 42.69% compared to 2000.

From the perspective of counties, the carbon sinks of the counties (districts) in Shaanxi Province are quite different, and the overall regional distribution of carbon sinks is characterized by “southern Shaanxi the most, northern Shaanxi second, and less in the middle”, as shown in Figure 3. Southern Shaanxi (Hanzhong City, Ankang City, Shangluo City) contains Ningqiang County, Liuba County, Yang County, Foping County, Xixiang County, Zhenba County, Shiquan County, Langao County, Xunyang County, Zhashui County, Shangluo County; the carbon sinks of these 11 counties (districts), including prefectures and districts, are at the highest level. The main reason for this is that southern Shaanxi has more precipitation, better initial resource endowment, stronger natural background restoration ability, and a higher survival rate of trees. As a result, the quality of forest stands is better, the stock of forest per unit area is higher. Moreover, the amount of carbon collection is higher under the implementation of key forestry projects. Although the initial carbon sinks in northern Shaanxi (Yan’an City and Yulin City) were relatively low, they were mostly in semi-arid areas with few natural forests. However, due to the strong implementation of key forestry projects, investment in the SLCP in northern Shaanxi alone reached 13.892 billion yuan from 2000 to 2015. Among all counties, Zhidan County, Wuqi County, Zichang County, Ansai County, and other counties invested more than 876 million yuan, and most of them are ecological forests. Therefore, although northern Shaanxi has low rainfall and poor resource endowments, its carbon sinks grew at a faster rate. Among the norther counties, Fuxian, Hengshan County and most counties in Yan’an City grew rapidly, and their total carbon sink amount reached the level of some counties (districts) in southern Shaanxi.

### 3.2. Analysis of Spatial Distribution Direction

To calculate the center-of-gravity migration trajectory of the carbon sinks in Shaanxi Province, this paper used the spatial statistical tools in ArcGIS 10.7 software to visualize the center-of-gravity migration trajectory from 2000 to 2017 (Figure 4). The statistical parameters of the SDE of carbon sinks in each county (district) each year were calculated (Table 1).

From 2000 to 2017, the center of gravity of carbon sinks in Shaanxi Province showed a characteristic of “south to north” migration, and the center of gravity of carbon sinks moved 35.89 km to the south. Among them, the distance moved significantly in 2000–2002 and 2008–2014, and the center of gravity in the two periods moved 16.66 km and 17.01 km, respectively. From 2000 to 2017, the area of the carbon sink ellipse showed a fluctuating upward trend. The overall area of the carbon sink ellipse increased by 2407.41 km^2^, with a growth rate of 1.95%. The area of the ellipse reached its maximum value in 2016 at 126,644.75. The results of the shift in the center of gravity and the area of the ellipse were consistent with the results of the aforementioned analysis of the spatiotemporal evolution of carbon sinks in Shaanxi Province. First, the result from the above-mentioned spatiotemporal evolution was that the carbon sinks in northern Shaanxi increased in both value and speed. Therefore, the center of gravity of carbon sinks in Shaanxi moved to the north. The main reasons are that the SLCP in northern Shaanxi and the NFPP were heavily invested in; additionally, the basic carbon sinks in northern Shaanxi are relatively low, and there is more room for increasing carbon sinks. Second, due to the increase in the overall vegetation coverage of Shaanxi Province, the overall area of the ellipse is showing signs of increase.

Specifically, from 2014 to 2017, the area of the carbon sink SDE fluctuated slightly, and the area of the ellipse decreased in 2014–2015 and 2016–2017. The possible reason for this is that from the perspective of the growth cycle of the trees, the annual net productivity of the main afforestation tree species, such as pine and acacia, showed an increase first, and then a downward trend after reaching the maximum. Therefore, it may cause a decline in regional carbon sinks. The length of the short axis of the Shaanxi carbon sink SDE was continuously shortening, while the length of the long axis constantly increased. The standard deviation of the X-axis decreased year by year, and decreased by 5.02% from 2000 to 2017; the standard deviation of the Y-axis increased year by year, increasing from 279,080.44 m in 2000 to 294,172.67 m in 2017.

Overall, the effect of increasing carbon sinks in Shaanxi Province was obvious, and the trajectory of the overall carbon sink’s center of gravity shifted northward. However, in the latter part of the study period (2014–2017), the area of the SDE of carbon sinks decreased, and the center of gravity of carbon sinks moved slightly to the south.

### 3.3. Analysis of Spatial Pattern Characteristics

(1) It can be seen from Table 2 that the global Moran’s I index of carbon sinks in Shaanxi Province from 2000 to 2017 showed an overall upward trend, ranging from 0.635 to 0.772, and all passed the 1% significance level test. It shows that the carbon sinks of Shaanxi Province are not randomly distributed in geographical space, but have significant positive global spatial autocorrelation. Specifically, there were small decreases in 2003, 2005, 2011, 2015, and 2017, indicating that the global spatial correlation of carbon sinks in Shaanxi Province gradually increased during the study period, though there is a small degree of volatility. Through the results of the global spatial autocorrelation test, it was found that the basic assumptions, based on the mutual independence of samples in traditional research, are not consistent with reality, and spatial effects need to be incorporated into the model.
(2) According to the analysis in Figure 5, the characteristics of the local spatial pattern of carbon sink levels in Shaanxi Province from 2000 to 2017 can be classified into four categories. The first type is the high-value agglomeration type (HH); this type indicates that the local county (district) has a high carbon sink level, and its surrounding counties (districts) also have a high carbon sink level, showing a spatially associated agglomeration state of “high center and high surroundings”. The second type is the high-value convex type (HL); this type means that the local county (district) has a high carbon sink level, but its surrounding counties (districts) have a low carbon sink level, showing the characteristics of “high in the center and low in the surrounding area”, and it is a state of spatial non-equilibrium correlation aggregation. The third type is the low-value collapse type (LH), which means that the local county (district) has a low carbon sink level, but its neighboring counties (districts) have a high carbon sink level, showing the characteristics of “low center and high surroundings”, and it is a state of spatial non-equilibrium correlation aggregation. The fourth type is the low-value agglomeration type (LL); this type indicates that the local county (district) has a low carbon sink level, and its neighboring counties (districts) also have a low carbon sink level, showing the characteristics of “low center and low surroundings”, and it is a low-level average spatial correlation agglomeration state.

Specifically, the carbon sink level of each county (district) in Shaanxi Province during the study period is mainly low-value agglomeration and high-value agglomeration, supplemented by high-value bulges and low-value subsidence. The trends of the four types of agglomeration have strong path dependence, and there are roughly parallel trends, with small fluctuations, among the types. As the carbon sinks of the counties (districts) in Shaanxi Province have neighboring peer effects and spatial spillover effects in geographic space, it is easier to form a local spatial pattern feature of “lower is always low, and higher is always high”.

### 3.4. Analysis of Influencing Factors of Carbon Sinks

#### 3.4.1. Spatial Measurement Model Inspection and Selection

The spatial measurement model includes three models: spatial error model (SEM), spatial lag model (SLM), and spatial Durbin model (SDM). Each model can correspond to no fixed effect, a space fixed effect, a time fixed effect, and a space-time double fixed effect. However, in actual use, a series of tests are required to select a model that matches the research question.

(1) LM test: examine the LM and test results without fixed effects, spatial fixed effects, temporal fixed effects, or spatio-temporal double fixed effects. It can be seen from Table 3 that in the LM test, both the LM-lag and LM-error tests passed the 1% significance level test. However, in robust LM test, the LM-lag test failed the 10% significance level test (*p* = 0.269), while the LM-error test passed the 1% significance level test. This indicates that the spatial lag model and the spatial error model may exist at the same time, and the spatial Doberman model is preferred.

(2) LR-Wald test and Hausman test: The LR-Wald test is to examine whether the spatial Doberman model can be degenerated into a spatial lag model or a spatial error model [33]. The results are shown in Table 4. The LR test and Wald test both reject the null hypothesis that the spatial Durbin model can be degenerated into a spatial lag model and a spatial error model at the 1% significance level. Therefore, we should choose the spatial Durbin model. At the same time, the Hausman test rejects the null hypothesis of random effects at the 1% significance level, so the fixed-effect spatial panel Durbin model should be selected.

(3) Selection of the spatial panel Durbin model: Table 5 shows the estimated results of the spatial panel Durbin model under space fixed effects, time fixed effects, and space-time double fixed effects. The analysis shows that the spatial regression coefficient ρ of the explained variable is positive under the three fixed effect estimates, all passed the 1% significance level test, and the results are relatively robust. This proves that the carbon sinks in Shaanxi Province have a significant positive spatial spillover effect, and the carbon sinks of each county (district) have a significant positive impact on the carbon sinks of other neighboring counties (districts). It further shows that it is reasonable and necessary to incorporate spatial effects into the measurement model when analyzing the influencing factors of the carbon sinks of the counties (districts) in Shaanxi Province.

The optimal fixed effects model was selected by comparing the R-squared, log-likelihood, and sigma^2^ values of the three models. The criteria for selecting the model were the largest R-squared and log-likelihood values, and the smallest sigma^2^ value. Among them, the R^2^ of the space-time dual fixed effects was 0.9910, which was greater than the other two models; the maximum value of log-likelihood was 112.44549, which corresponded to the space-time dual fixed effects model. However, the minimum value of sigma^2^ was 0.0470, which corresponds to the spatial fixed-effects model. After comparison, the sigma^2^ value of the space-time dual fixed effects model was only 0.0001 higher than that of the space fixed effect, but the log-likelihood value was approximately 20.85 higher than that of the space fixed effect model. Based on the above test results, this paper finally chose the space-panel Durbin model, with double fixed effects in space and time. However, the coefficients of the explanatory variables in the spatial Durbin model in Table 6 do not directly represent marginal effects, nor do they represent direct effects or spatial spillover effects. Therefore, this was different from the traditional method of analyzing regression coefficients of non-spatial models, which have no actual economic meaning.

#### 3.4.2. Spatial Effect Decomposition Analysis

Table 6 shows the decomposition results of the spatial effects of various influencing factors of carbon sinks based on the spatial panel Durbin model under the dual fixed effects of time and space.

(1) The direct population effect coefficient was −0.0015, but it did not passed the significance test. The population spillover effect coefficient was −0.0180, and it passed the 5% significance level test. This shows that the increase of local population level has not yet formed a significant promotion effect on the increase of local carbon sinks, but it limits the increase of carbon sinks in neighboring areas through negative spillover effects. The main reason for this is that the increase in local population may have a siphon effect on neighboring counties (districts) [35]. That is, as the local population increases, the population of neighboring counties (districts) flows out accordingly. Corresponding resources and policies tilt towards the local area, and the forestland management intensity and carbon sink demand of neighboring counties (districts) is reduced, which limits the level of carbon sinks in neighboring counties (districts).
(2) The direct effect coefficient of per capita GDP on carbon sinks was 0.0220, and the spillover effect coefficient was −0.0434, and both passed the 1% significance level test. This shows that an increase of local GDP per capita promotes the increase of local carbon sink level, but restrains the increase of carbon sinks in neighboring counties (districts) through negative spillover effect. The main reason for this is that the improvement of the economic level of the region manifests as an increase of income and an increase in people’s requirements for quality of life. Therefore, the need to improve the environment and improve the greening rate increases. At the same time, some ecological projects of Zenghui attract and create employment opportunities, promote the development of tourism, improve local employment levels, and increase income. Therefore, there is a virtuous circle between the two. However, there is also a siphon effect in neighboring areas, which limits the level of carbon sinks in neighboring counties (districts).
(3) The direct effect coefficient of local government general budget revenue on carbon sinks was 0.0700, and the spillover effect coefficient was 0.3157, and both passed the 1% significance level test. This shows that an increase in the general budget level of local governments not only promotes the improvement of local carbon sinks, but also promotes the increase of carbon sinks in neighboring counties (districts) through positive spillover effects. Increasing forest area and improving forest quality require financial support from the local government. The advancement of projects, such as the SLCP and NFPP, also requires continuous investment of government funds. Therefore, the increase of carbon sinks needs to rely on financial subsidies from the local government to a certain extent, so an increase in the general budget revenue of local governments has a positive effect on local carbon sinks. Additionally, due to the comparison of environmental performance assessment and evaluation among county (district) governments, it has a certain incentive effect on surrounding counties (districts).
(4) The direct effect coefficient of local government general budget expenditures on carbon sinks was −0.0945, and the spillover effect coefficient was −0.6211, and both passed the 1% significance level test. This shows that the increase of local government’s general budgetary expenditure limits the level of local carbon sinks, and restrains neighboring counties (districts) from increasing carbon sinks through negative spillover effects. An increase in general budgetary expenditures of local governments in the study area may be used for education, social security and employment, medical and health care, urban and rural community affairs, and transportation. Therefore, the urbanization development of the region is accelerated, and the space for new forestation is restricted.
(5) The direct effect coefficient of total retail sales of social consumer goods carbon sink was −0.0179, and the spillover effect coefficient was 0.0084, but neither of them passed the significance level test. The reason for these insignificant results may be that the total retail sales of consumer goods mainly reflects the consumption needs of residents in the region. The increase in carbon sinks is mainly related to government policies and has no obvious direct correlation with residents’ consumption levels.

## 4. Discussion

This paper used panel data from 107 counties (districts) in Shaanxi Province from 2000 to 2017. First, we comprehensively used ESDA, spatial distribution directional analysis, and the spatiotemporal evolution of carbon sinks to analyze the distribution pattern and evolution of carbon sinks in Shaanxi Province, to explore the internal mechanisms. Then, the spatial Durbin model was selected to investigate the influencing factors of carbon sink changes. Analysis of the results showed that the increase or decrease of carbon sinks in Shaanxi province’s counties (districts) was affected by population, per capita GDP, local government general budget expenditures, and local government general budget revenues. The main differences from previous studies were:

(1) Visualization of the increase and decrease of carbon sinks in each county (district) in Shaanxi Province in time and space, so as to facilitate dynamic analysis.
(2) Visualization of the migration trajectory of the center of gravity of carbon sinks in Shaanxi Province from 2000 to 2017, and use of the SDE to reflect the changes in its spatial distribution pattern.
(3) Use of ESDA to analyze the global and local spatial autocorrelation of carbon sinks, and construction of a spatial weight matrix to use the spatial Durbin model, avoiding the endogenous problem of model settings.

The main significant aims of this paper were to analyze the impact mechanism of carbon sinks at the county level, and construct a spatial weight matrix to incorporate geographic factors into the research system. At the same time, the spatiotemporal evolution of carbon sink distribution in Shaanxi Province was visualized to the greatest extent. The research results provided a decision-making basis for the allocation of funds by county (district) governments regarding high-quality forest development and the realization of the goal of “carbon neutrality” in the region.

During the study period, the temporal changes of carbon sinks in Shaanxi Province showed an overall upward trend. The possible reason for this lies in the implementation of the SLCP and the NFPP in Shaanxi Province. In 1999, the SLCP was implemented as a pilot in Shaanxi Province. As of 2018, total investment in the SLCP has exceeded 500 billion yuan, and the SLCP has increased the area of forestland by 3346.67 × 104 hm^2^, accounting for 42.5% of the planted forest area. So far, Shaanxi Province has completed the SLCP with an area of 24,370 km^2^ [36]. The NFPP has been implemented in Shaanxi Province for two phases since 1998. Each phase reduced the consumption of forest resources by more than 5.5 million m^3^ each year. As of 2010, the forest resource consumption in Shaanxi Province was reduced from 9.466 million m^3^ to 2.376 million m^3^, a decrease of 74.9%, and the forest coverage rate in Shaanxi Province increased from 30.92%, before the implementation of the two projects, to 43.06% [36]. The increase in large-scale forestry area and the decrease in forest resource consumption greatly improved the overall forest carbon sequestration capacity.

Previous research methods did not consider the spatial interaction and spatial unevenness of the panel data in the model. Moran’s I statistics showed that the spatial distribution of carbon sinks in the counties (districts) of Shaanxi Province was not random, but that there was a significant positive global spatial autocorrelation. Therefore, the use of traditional measurement models would cause the endogenous problems of the model to cause errors in the estimation results, and fail to provide effective policy recommendations [37]. This paper analyzed the results of the spatial panel Durbin model of the dual fixed effects of carbon sinks in time and space. The existence of the spatial spillover effect of carbon sinks determined that county-level governments should not only consider the situation in their jurisdictions in the process of increasing carbon sinks. In the context of regional integrated development, the spatial interaction between neighboring regions in the region should be considered comprehensively, and the linkage-management and control roles of counties (districts) in increasing carbon sinks should be strengthened. 

Of course, our research also has limitations. Our research area was limited to Shaanxi Province, and the research results have specific policy significance for 107 counties (districts). However, due to regional heterogeneity, whether the spatial measurement model is applicable to carbon sinks in other regions, and whether the impact mechanism of carbon sinks is consistent in other regions, remains unverified. Future research should explore the path of regional carbon neutrality, and seek the most cost-effective carbon neutral path based on the principle of cost-effectiveness.

## 5. Conclusions

This paper used the panel data of 107 counties (districts) in Shaanxi Province, from 2000 to 2017, as the research sample. First, we conducted spatial distribution directional analysis and ESDA of carbon sinks. Then, we constructed the geographic spatial weight matrix, and used the spatial panel Durbin model to analyze the driving factors of carbon sink changes in Shaanxi Province from the perspective of spatial effects. 

The main conclusions of the study were:

(1) The temporal change of carbon sinks in Shaanxi Province showed an overall upward trend. In 2017, carbon sinks increased by 42.69%, compared with 2000. Counties (districts) differed greatly in carbon sinks, showing the characteristics of “southern Shaanxi is the most, northern Shaanxi is the second, and the middle is few”.
(2) The center of gravity of carbon sinks in Shaanxi Province as a whole showed the characteristics of “south to north” migration, and the center of gravity of carbon sinks moved 35.89 km to the south, as a whole. The area of the carbon sink SDE showed a fluctuating upward trend, and the growth rate from 2000 to 2017 was 1.95%. Among them, the area of the SDE from 2014 to 2017 fluctuated slightly.
(3) There was a significant positive global spatial autocorrelation of carbon sinks in Shaanxi Province in geographic space. The overall Moran’s I index showed a significant upward trend, though there were small fluctuations. The local pattern was characterized by low-value agglomeration and high-value agglomeration, supplemented by high-value bulge and low-value collapse. The trends of the four types of agglomeration had strong path dependence, and there were roughly parallel trends with small fluctuations among each type.
(4) Per capita GDP and general budget revenue of local governments had a positive direct effect on the level of local carbon sinks. The general budget expenditures of local governments had a negative direct effect on the level of local carbon sinks. The general budget revenue of local governments had a positive spillover effect on the carbon sink level of neighboring counties (districts). Population, GDP per capita, and general budget expenditures of local governments had a negative spillover effect on the carbon sink level of neighboring counties (districts).

We propose the following policy suggestions according to the research conclusions: First, the government should start from the source of pollution and strengthen the control of carbon emissions from key industries. Enterprises should use technological innovation as a means to promote the renewal and upgrade of environmental protection equipment in heavily polluting enterprises. Second, in the process of advancing the realization of “carbon neutrality”, the spatial interaction between neighboring areas in the region should be comprehensively considered. Counties (districts) should cooperate with each other in the process of policy formulation and implementation to ensure the environmental benefits of various forestry projects. Third, the government should seek the most cost-effective way to increase carbon sinks. Both short-term and long-term goals need to be taken into consideration, as well as the coordination and unity of environmental benefits, economic benefits, and social benefits to promote regional sustainable development.

## Figures and Tables

**Table 1:** Changes in the SDE of carbon sinks.

Year	Shape Area/km^2^	Center X/m	Center Y/m	XStdDist/m	YStdDist/m	Rotation/°
2000	123,265.80	326,788.56	3,672,245.62	140,605.88	279,080.44	15.39
2001	123,746.70	326,640.94	3,674,453.67	140,804.95	279,773.16	15.02
2002	124,630.18	328,875.28	3,687,475.04	139,086.02	285,254.27	14.98
2003	123,204.19	329,356.78	3,689,104.61	138,433.44	283,319.66	15.61
2004	123,547.09	330,222.57	3,689,850.28	138,095.94	284,802.87	15.44
2005	123,267.13	327,009.68	3,685,458.52	139,221.28	281,859.85	15.46
2006	122,141.66	331,506.39	3,689,306.27	138,636.00	280,465.41	15.15
2007	122,771.62	330,504.07	3,688,084.10	138,584.97	282,015.99	15.30
2008	123,186.31	330,686.29	3,691,118.70	138,260.35	283,633.29	15.18
2009	123,733.75	330,065.74	3,692,530.05	138,043.11	285,342.43	15.17
2010	124,208.95	330,459.81	3,696,991.01	137,204.39	288,189.98	15.29
2011	124,051.09	329,424.10	3,696,805.91	137,287.04	287,650.31	15.64
2012	123,814.28	331,733.14	3,702,979.71	136,466.94	288,827.02	15.44
2013	125,449.62	328,801.24	3,703,514.55	136,436.06	292,708.72	15.47
2014	126,039.59	330,626.75	3,708,144.62	135,562.50	295,981.25	15.30
2015	123,252.07	330,368.15	3,696,889.53	137,147.83	286,087.45	15.33
2016	126,644.75	332,152.13	3,708,693.15	136,511.19	295,335.08	15.18
2017	125,673.21	331,553.43	3,707,378.05	135,999.24	294,172.67	15.34

**Table 2:** Moran’s I test results.

Year	Moran I	Z-Score	*p* Value
2000	0.635	11.942	0.000
2001	0.640	12.047	0.000
2002	0.697	13.072	0.000
2003	0.684	12.848	0.000
2004	0.708	13.288	0.000
2005	0.676	12.701	0.000
2006	0.702	13.168	0.000
2007	0.702	13.170	0.000
2008	0.713	13.380	0.000
2009	0.718	13.471	0.000
2010	0.736	13.805	0.000
2011	0.733	13.737	0.000
2012	0.746	13.987	0.000
2013	0.753	14.110	0.000
2014	0.772	14.465	0.000
2015	0.732	13.722	0.000
2016	0.770	14.425	0.000
2017	0.767	14.353	0.000

**Table 3:** LM test results.

Model	No Space Lag		No Spatial Error	
	LM (*p*-Value)	R-LM (*p*-Value)	LM (*p*-Value)	R-LM (*p*-Value)
No fixed effect	1016.0244 (0.000)	22.2761 (0.000)	1022.5884 (0.000)	28.8401 (0.000)
Spatial fixed effects	1420.3354 (0.000)	13.6961 (0.000)	1900.4270 (0.000)	493.7877 (0.000)
Time fixed effect	958.7425 (0.000)	29.0455 (0.000)	978.6383 (0.000)	48.9413 (0.000)
Space-time dual fixed effect	1216.0203 (0.000)	1.2198 (0.269)	1454.4090 (0.000)	239.6086 (0.000)

**Table 4:** Results of diagnosis and selection test of spatial measurement model.

Testing Method	Spatial Fixed Effects	Time Fixed Effect	Space-Time Dual Fixed Effect
LR test spatial lag	30.6405 ***	129.4123 ***	31.9249 ***
Wald test spatial lag	29.2109 ***	132.9749 ***	30.7833 ***
LR test lag spatial error	45.0008 ***	103.1242 ***	19.0978 ***
Wald test spatial error	42.7525 ***	105.1958 ***	15.9113 ***
Hausman	7193.5316 ***	545.3648 ***	-

Note: *** indicates significance at the significance level of 1%.

**Table 5:** Estimation results of the spatial panel Durbin model.

Variable	Spatial Fixed Effects		Time Fixed Effect		Space-Time Dual Fixed Effect	
	Coef.	*t*-Stat.	Coef.	*t*-Stat.	Coef.	*t*-Stat.
population	0.0006	0.5071	0.0183 ***	7.9808	−0.0001	−0.0928
agdp	0.0267 ***	5.4137	0.1166 ***	4.8157	0.0256 ***	5.1676
lnincome	0.0351 *	1.8029	−0.4142 ***	−5.9704	0.0459 **	2.3188
lnoutcome	−0.0269	−1.0169	1.0349 ***	11.9747	−0.0463 *	−1.7067
lnretail	−0.0173	−1.5148	−0.4262 ***	−7.8622	−0.0191 *	−1.6518
W × population	−0.0019	−0.9821	−0.0360 ***	−7.7888	−0.0049 **	−2.3762
W × agdp	−0.0318 ***	−4.6103	−0.1515 ***	−4.3364	−0.0311 ***	−4.3880
W × lnincome	−0.0013	−0.0470	0.5904 ***	5.5074	0.0541	1.5907
W × lnoutcome	0.0431	1.3908	−0.9540 ***	−5.9891	−0.1408 ***	−2.6438
W × lnretail	0.0315	1.5265	0.1050	1.0820	0.0157	0.7076
*ρ*	0.7790 ***	52.4570	0.6560 ***	34.0420	0.7427 ***	45.3354
sigma^2^	0.0470		2.1810		0.0471	
Log-likelihood	91.591922		−3595.055		112.44549	
R-squared	0.9909		0.5580		0.9910	

Note: ***, ** and * indicate significance at the significance level of 1%, 5% and 10% respectively.

**Table 6:** Spatial effect decomposition results of the spatial panel Durbin model.

Variable	Direct Effect		Spillover Effect		Total Effect	
	Coef.	*t*-Stat.	Coef.	*t*-Stat.	Coef.	*t*-Stat.
population	−0.0015	−1.0570	−0.0180 **	−2.5108	−0.0195 **	−2.4440
agdp	0.0220 ***	4.3596	−0.0434 **	−2.2226	−0.0214	−1.0135
lnincome	0.0700 ***	3.1070	0.3157 ***	2.6820	0.3857 ***	2.9770
lnoutcome	−0.0945 ***	−2.9547	−0.6211 ***	−3.3572	−0.7155 ***	−3.5022
lnretail	−0.0179	−1.2069	0.0084	0.1020	−0.0095	−0.1029

Note: *** and ** indicate significance at the significance level of 1% and 5% respectively.

## Data Availability

The data presented in this study are available in this article.
